# Systematic Approach to the Diagnosis and Treatment of Lyme Carditis and High-Degree Atrioventricular Block

**DOI:** 10.3390/healthcare6040119

**Published:** 2018-09-22

**Authors:** Cynthia Yeung, Adrian Baranchuk

**Affiliations:** Department of Medicine, Queen’s University, Kingston, ON K7L 3N6, Canada; cyeung@qmed.ca

**Keywords:** Lyme disease, Lyme carditis, atrioventricular block

## Abstract

Lyme carditis (LC) is a manifestation of the early disseminated stage of Lyme disease and often presents as high-degree atrioventricular (AV) block. High-degree AV block in LC can be treated with antibiotics, usually resolving with a highly favorable prognosis, thus preventing the unnecessary implantation of permanent pacemakers. We present a systematic approach to the diagnosis and management of LC that implements the Suspicious Index in Lyme Carditis (SILC) risk stratification score.

## 1. Introduction

Lyme disease is an infection that can manifest in a multi-system nature, usually transmitted by the tick *Ixodes scapularis* and caused by a Gram-negative spirochete bacteria, *Borrelia burgdorferi*. The most commonly reported vector-borne disease in North America [1], Lyme disease presents mainly between March and October, with over 60% of reported cases in June and July [2].

Lyme carditis (LC) was first described in 1980 by Steere, et al. and is an early manifestation of Lyme disease, appearing within one to two months (range < 1 to 28 weeks) after the onset of infection [3,4]. The incidence of cardiac involvement in Lyme disease is estimated to be 0.3% to 4% [5]. Compared to the relatively equal prevalence of Lyme disease by sex, LC has a strong male predominance of approximately 3:1 [2]. Although other asymptomatic conduction disorders are possible—including sinus node disease, intraatrial block, abnormal nodal recovery time, and interventricular delay [5,6,7,8,9]—high-degree atrioventricular (AV) block is the most common, in approximately 90% of LC, and requires cardiac monitoring [3,7].

The transmural inflammation in LC is predominantly comprised of macrophages and lymphocytes [10], and is concentrated at the base of the heart, the basal interventricular septum, and the perivascular regions [10,11]. The pathophysiology of AV node involvement in LC may be explained by its anatomical location, histology, and metabolic mechanisms [12]. The block is often above the bundle of His at the AV node level [4]. Patients with a PR interval > 300 milliseconds are at the highest risk for progression to complete AV block [4]. Progression to complete heart block can be rapid, and if untreated, potentially fatal [13,14,15].

In general, the treatment for a high-degree AV block is pacing. However, the AV block in LC may revert back to normal conduction, and usually resolves within the first 10 days of antibiotic administration [12,16,17]. If the AV block in LC is indeed transient, then a permanent pacemaker is not indicated [16]. Therefore, the identification of LC in patients with a high-degree AV block is imperative to prevent the inherent risks of pacemaker implantation, such as periprocedural infections, lead dislodgement, and erosions [18]. Furthermore, given the young demographic of patients with LC, unnecessary pacemaker implantation would result in the subsequent lifetime of multiple pulse generator changes and burden of associated cumulative health care costs [19].

## 2. Systematic Approach to the Diagnosis and Management of Lyme Carditis

Case series have demonstrated that patients often present several times before the LC is suspected [19]. Many patients with LC do not recall a clear history of a tick bite. Although erythema migrans is present in 70–80% of Lyme disease cases [6,20], the pathognomonic rash is less common (40%) in LC [5]. Prompt treatment of LC shortens the duration of the cardiac manifestations and prevents later complications of Lyme disease [5,21]. Failure to recognize and treat Lyme disease aggressively in its early stages may increase the need for temporary or permanent pacing [17].

### 2.1. The Suspicious Index in Lyme Carditis (SILC) Risk Score

In order to evaluate the likelihood that a patient’s high-degree heart block is caused by LC, we proposed the Suspicious Index in Lyme Carditis (SILC) score (Table 1) [22]. This novel risk score assigns weights to several risk factors: age < 50 [5,6,19]; male sex [2]; outdoor activity or endemic area [2,23]; constitutional symptoms of Lyme disease, including fever, malaise, arthralgia, dyspnea, pre-syncope, and syncope [4,24]; history of a tick bite [19]; and erythema migrans [6]. A preliminary validation study in which the SILC risk stratification tool was retrospectively applied to 88 cases of LC (83 from a systematic review of all published cases of LC with high degree AV block and five from our own experience) demonstrated a sensitivity of 93.2% (if a variable was not available, it was conservatively assigned a zero). The sensitivity increased to 100% when the SILC score was applied to cases that reported on all SILC variables (*n* = 32) [22].

### 2.2. Algorithm for the Diagnosis and Management of Lyme Carditis

**Figure 1:**
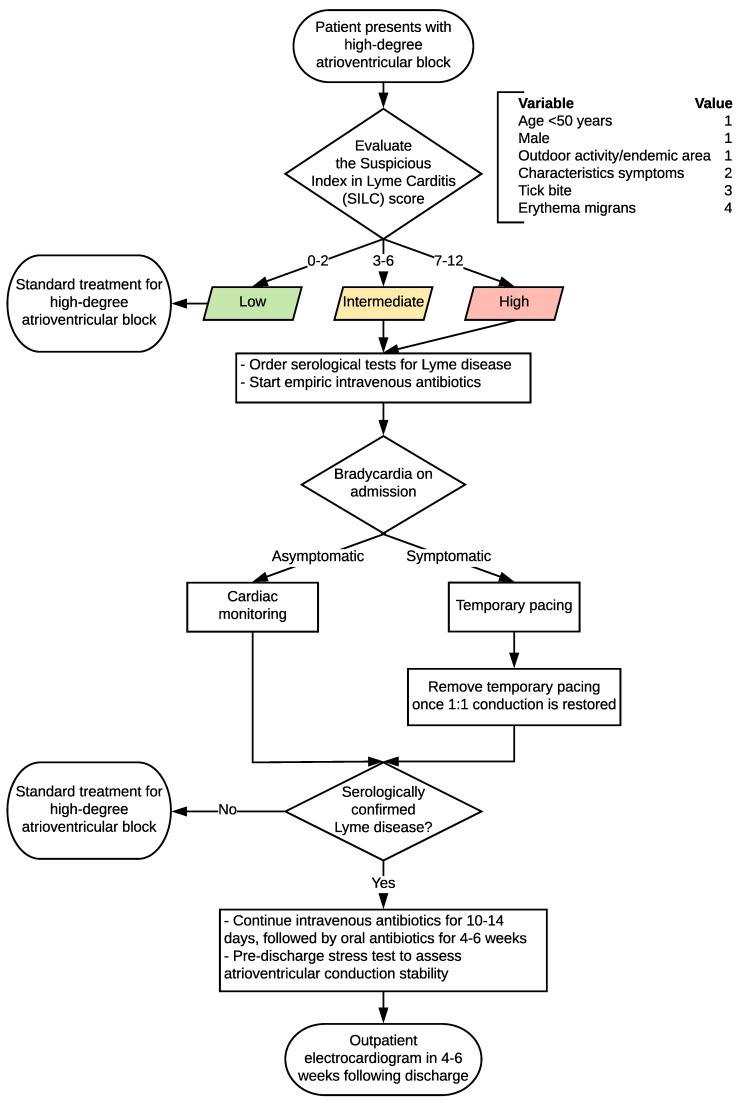
Systematic approach to the diagnosis and management of Lyme carditis and high-degree atrioventricular block.

A flowchart summarizing our algorithm for the systematic approach to the diagnosis and management of LC is presented in Figure 1. The SILC score should be calculated for the patient presenting with a high-degree AV block. If the summed SILC score is 0–2 (low risk), then standard treatment for a high-degree AV block should be followed. If the summed SILC score is 3–6 (intermediate risk) or 7–12 (high risk), serological tests for Lyme disease (positive enzyme-linked immunosorbent assay (ELISA) and Western blot) should be ordered to confirm the infection [25,26,27].

Blood serologies can be falsely negative due to the delayed immune response, and consequently, negative serology does not always rule out early Lyme infection. However, since LC is a manifestation of the disseminated stage of Lyme disease, the vast majority of patients with LC have positive serologic responses with either IgM and/or IgG antibodies [28]. Notably, serum IgG antibodies may be present long after recovery from Lyme disease, and thus, seropositivity may not translate to a recent *B. burgdorferi* infection being the cause of a cardiac presentation.

While the results of the Lyme serology are being processed, empiric intravenous (IV) antibiotics should be started [21]. Despite the lack of comparative trials on the optimal antibiotic regimen or route for LC, expert opinion and supportive data from case reports indicate that ceftriaxone (2 g IV once daily in adults; 50–75 mg/kg IV once daily in children) is first-line therapy, but appropriate alternatives include IV cefotaxime or penicillin G.

Management for bradycardia on admission is dependent on whether it is symptomatic. Asymptomatic bradycardia should be followed with cardiac monitoring, because patients with LC can progress from a prolonged PR interval to complete block and asystole, and the degree of AV block can fluctuate rapidly [29,30]. Symptomatic bradycardia should be managed with temporary pacing (standard or temporary-permanent pacemaker). Approximately one-third of patients with LC require temporary pacing [3,16,19].

If Lyme disease is serologically confirmed, IV antibiotics should be continued for 10–14 days, followed by a four to six weeks oral antibiotic regimen. Appropriate oral antibiotics include doxycycline, amoxicillin, and cefuroxime axetil (doxycycline should not be used in pregnant women or children <8 years). A pre-discharge stress test should be ordered to assess the stability of atrioventricular conduction. An outpatient electrocardiogram arranged for four to six weeks post-discharge is appropriate to confirm a normal PR interval and the lack of any other rhythm or conduction abnormalities.

## 3. Conclusions

The SILC risk score may help identify LC in patients presenting with high-degree AV block. High-degree AV block in LC can be treated with antibiotics, often resolving with a highly favorable prognosis, thus preventing the unnecessary implantation of permanent pacemakers.

## Figures and Tables

**Table 1:** The Suspicious Index in Lyme Carditis (SILC) score evaluates the likelihood that a patient’s high-degree heart block is caused by Lyme carditis. The total summed score indicates low (0–2), intermediate (3–6), or high (7–12) suspicion of Lyme carditis.

Variable	Value
Age < 50 years	1
Male	1
Outdoor activity/endemic area	1
Constitutional symptoms ^1^	2
Tick bite	3
Erythema migrans	4

^1^ Fever, malaise, arthralgia, dyspnea, pre-syncope, and syncope.
